# Neurotensin receptor 1 signaling promotes pancreatic cancer progression

**DOI:** 10.1002/1878-0261.12815

**Published:** 2020-11-20

**Authors:** Kei Takahashi, Shogo Ehata, Kensuke Miyauchi, Yasuyuki Morishita, Keiji Miyazawa, Kohei Miyazono

**Affiliations:** ^1^ Department of Molecular Pathology Graduate School of Medicine The University of Tokyo Bunkyo‐ku Japan; ^2^ Environmental Science Center The University of Tokyo Bunkyo‐ku Japan; ^3^ Department of Biochemistry Graduate School of Medicine University of Yamanashi Chuo Japan

**Keywords:** neurotensin, NTSR1, orthotopic inoculation model, pancreatic cancer, SR48692

## Abstract

Pancreatic cancer is one of the cancers with the poorest prognosis, with a 5‐year survival rate of approximately 5–10%. Thus, it is urgent to identify molecular targets for the treatment of pancreatic cancer. Using serial transplantations in a mouse pancreatic orthotopic inoculation model, we previously produced highly malignant pancreatic cancer sublines with increased tumor‐forming abilities *in vivo*. Here, we used these sublines to screen molecular targets for the treatment of pancreatic cancer. Among the genes with increased expression levels in the sublines, we focused on those encoding cell surface receptors that may be involved in the interactions between cancer cells and the tumor microenvironment. Based on our previous RNA‐sequence analysis, we found increased expression levels of neurotensin (NTS) receptor 1 (NTSR1) in highly malignant pancreatic cancer sublines. Furthermore, re‐analysis of clinical databases revealed that the expression level of NTSR1 was increased in advanced pancreatic cancer and that high NTSR1 levels were correlated with a poor prognosis. Overexpression of NTSR1 in human pancreatic cancer cells Panc‐1 and SUIT‐2 accelerated their tumorigenic and metastatic abilities *in vivo*. In addition, RNA‐sequence analysis showed that MAPK and NF‐κB signaling pathways were activated upon NTS stimulation in highly malignant cancer sublines and also revealed many new target genes for NTS in pancreatic cancer cells. NTS stimulation increased the expression of MMP‐9 and other pro‐inflammatory cytokines and chemokines in pancreatic cancer cells. Moreover, the treatment with SR48692, a selective NTSR1 antagonist, suppressed the activation of the MAPK and NF‐κB signaling pathways and induction of target genes in pancreatic cancer cells *in vitro*, while the administration of SR48692 attenuated the tumorigenicity of pancreatic cancer cells *in vivo*. These findings suggest that NTSR1 may be a prognostic marker and a molecular target for pancreatic cancer treatment.

## Graphical abstract

Neurotensin (NTS) receptor 1 (NTSR1) is expressed in highly malignant pancreatic cancer cells, which were established by serial orthotopic transplantations. NTS/NTSR1 signaling contributes to the progression of pancreatic cancer through the activation of MAPK and NF‐κB signaling pathways and the induction of the genes related to inflammation. Targeting NTSR1 signaling by SR48692 inhibits pancreatic cancer progression.

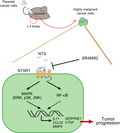

**Abbreviations**
MAPK: mitogen‐activated protein kinase
NF‐κB: nuclear factor‐κB
NTS: neurotensin
NTSR1: neurotensin receptor 1
qRT–PCR: quantitative real‐time reverse transcription–PCR
RNA‐seq: RNA‐sequence

## 1 Introduction

Diagnostic technologies and cancer treatments have developed drastically over the last couple of decades. However, the 5‐year survival rate of pancreatic cancer patients remains around 5–10% [1, 2]. Although surgical resection plays a critical role in the management of pancreatic cancer, less than 20% of pancreatic cancer patients are eligible for surgery [3]. Chemotherapy using gemcitabine and 5‐fluorouracil in the adjuvant setting has been shown to improve the survival rates of pancreatic cancer patients [4]. Furthermore, recent studies showed that immunotherapy is expected to improve the survival of pancreatic cancer patients and many trials are ongoing [5]. However, the lethality of pancreatic cancer is estimated to remain high in the next decade [3]. Thus, a detailed understanding of the molecular mechanisms underlying pancreatic cancer is essential for the development of novel therapeutic approaches.

Mutations of many oncogenes and tumor suppressor genes are frequently observed in pancreatic cancer. Notably, mutations in *KRAS*, *TP53*, *CDKN2A,* and *SMAD4* are reported to be involved in pancreatic cancer development [1, 6, 7]. In addition to these genetic alterations, interactions between cancer cells and the tumor microenvironment are thought to contribute to cancer progression [8]. Especially in pancreatic cancer, there is an active interaction between cancer cells and various kinds of stromal cells and extracellular matrix (ECM) proteins in the tumor microenvironment [1]. Furthermore, pancreatic cancer is frequently accompanied by fibrotic tissue proliferation [1, 9].

To mimic the interactions between cancer cells and the tumor microenvironment, orthotopic inoculation models have been used to study various types of cancers [10, 11, 12, 13]. In our previous study, we utilized orthotopic inoculation models for pancreatic cancer and found that the interactions with the tumor microenvironment were crucial for the acquisition of a malignant phenotype by pancreatic cancer cells [14]. Serial orthotopic transplantation models allowed us to establish highly malignant cancer sublines in combination with *in vivo* bioluminescence imaging. Using this model, we obtained highly malignant pancreatic cancer sublines from primary tumors of several human pancreatic cancer cells, including Panc‐1 and SUIT‐2 [14]. These highly malignant sublines exhibited increased tumorigenic and metastatic abilities *in vivo,* with decreased expression of E‐cadherin. Furthermore, the gene expression profiles of these sublines were distinct from those of the parental cells, suggesting that characterization of the sublines might lead to the identification of molecule(s) that are important for cancer progression. Based on gene expression profiles, the expression of the stem cell marker nestin was increased in cancer cells as a result of the interaction with the pancreatic microenvironments, which increased tumor formation and invasiveness. In this study, we focused our investigations on cell surface receptors that were highly expressed in these sublines and extracted neurotensin (NTS) receptors (NTSRs) as candidates. Although several reports showed that NTS/NTSR signaling is involved in the progression of pancreatic cancer [15, 16, 17], downstream signaling pathways for NTS were not fully uncovered based on the genome‐wide gene expression study. We identified target(s) for NTS/NTSR signaling in pancreatic cancer cells and also aimed to determine whether NTS/NTSR1 signaling pathways might be potential target(s) for pancreatic cancer treatment *in vivo*.

## 2 Methods

### 2.1 Reagents

The NTS peptide (4029‐v) (Peptide Institute. Inc., Osaka, Japan) was dissolved in distilled water (0.1 mm). SR48692 (SML0278; Sigma‐Aldrich, Merck Millipore, Burlington, MA, USA) was dissolved in dimethyl sulfoxide (DMSO; 10 mg·mL^−1^; Nacalai Tesque, Kyoto, Japan) and used *in vitro* at a final concentration of 10 μm.

### 2.2 Mouse tumor models

All experiments were approved by and carried out according to the guidelines of the Animal Ethics Committee of the Graduate School of Medicine, The University of Tokyo. In the orthotopic inoculation model, pancreatic cancer cells were inoculated into the pancreas of 6‐week‐old female BALB/c‐*nu/nu* (nude) mice (CLEA Japan Inc., Tokyo, Japan) under anesthesia, as previously described [14]. In the SR48692 administration model, Panc‐1‐3P cells were inoculated orthotopically (5 x 10^5^ cells/mouse) (day 0) and either SR48692 or DMSO was administered intraperitoneally as follows: days −1, 1–7, 10 mg·kg^−1^, and days 8–19, 5 mg·kg^−1^. SR48692 (10 mg·mL^−1^) was diluted at a ratio of 1: 20 in phosphate‐buffered saline (PBS). The excised tumors were fixed with a 4% formaldehyde solution, embedded into paraffin, and then prepared for sectioning.

### 2.3 Cell culture and establishment of sublines

Parental cells of the human pancreatic adenocarcinoma cell SUIT‐2 (Japanese Cancer Research Resource Bank, Osaka, Japan) and Panc‐1 (American Type Culture Collection, Manassas, VA, USA) were maintained in Dulbecco’s Modified Eagle’s Medium (Gibco, Thermo Fisher Scientific) containing 10% fetal bovine serum (FBS, Gibco), 50 U·mL^−1^ penicillin, and 50 mg·mL^−1^ streptomycin. Highly malignant cancer sublines (3P, 3L, and 3Liv cells) were established and maintained as previously described [14].

### 2.4 Establishment of stable transfectants expressing NTSR1 or mCherry

To establish NTSR1‐overexpressing cells, a lentiviral vector system was used as previously described [18]. The lentiviral vector system was kindly provided by Dr. Hiroyuki Miyoshi (deceased, formerly Keio University). The human *NTSR1* gene was cloned from Panc‐1‐3P cells and inserted into the entry vector pENTR201. As a control, the mCherry gene (originated from pmCherry; Clontech Laboratories, Inc., Mountain View, CA, USA) was inserted into the pENTR201 [19]. The recombination reaction between pENTR201 and CSII‐EF‐RfA (destination vector) was performed using the Gateway LR Clonase II enzyme (Invitrogen, Thermo Fisher Scientific). Then, 293FT cells (Invitrogen) were transfected with an expression vector (CSII‐EF‐NTSR1 or CSII‐EF‐mCherry), VSV‐G, the Rev‐expressing construct (pCMV‐VSV‐G‐RSV‐Rev), and a packaging vector (pCAG‐HIVgp). The culture supernatants were concentrated as lentiviral particles using the Lenti‐X Concentrator (Clontech, Shiga, Japan).

### 2.5 Cell proliferation assay

Cells were seeded into 96‐well plates and cultured for 2–5 d. At the indicated days, the number of living cells was determined using Cell Count Reagent SF (Nacalai Tesque). Absorbance at 450 nm and 595 nm (reference absorbance) was measured with a Model 680 Microplate Reader (Bio‐Rad) as previously described [20].

### 2.6 Bioluminescence imaging

*In vivo* bioluminescence imaging was performed as previously described [21]. For *ex vivo* bioluminescence imaging, resected lungs and livers were incubated with D‐luciferin (Promega, Madison, WI, USA) and the bioluminescence signal was measured with Night OWL II LB983 (Berthold Technologies, Bad Wildbad, Germany). The images were analyzed with the IndiGO2 software (Berthold Technologies). All values were shown as photons per second.

### 2.7 Immunoblotting

Immunoblotting was performed as previously described [18]. Cell lysates were extracted with a lysis buffer (1% Nonidet P‐40, 150 mm NaCl, and 20 mm Tris/HCl). To obtain the cytoplasmic and nuclear fractions, the NE‐PER™ Nuclear and Cytoplasmic Extraction Reagents (#787833; Thermo Fisher Scientific) were used according to the manufacturer’s instructions. The protein concentrations were measured with the BCA Protein Assay (Pierce, Thermo Fisher Scientific). Proteins were applied to SDS/polyacrylamide gel electrophoresis and transferred to a membrane. Anti‐NTSR1 (PA3‐214; Thermo Fisher Scientific), anti‐phospho‐p44/42 MAPK (Erk1/2) (#9101; Cell Signaling Technology, Danvers, MA, USA), anti‐ERK (#9102; Cell Signaling Technology), anti‐phospho‐p38 MAP kinase (#9211; Cell Signaling Technology), anti‐p38 (#9228; Cell Signaling Technology), anti‐phospho‐SAPK/JNK (#9251; Cell Signaling Technology), anti‐p65 (#8242; Cell Signaling Technology), anti‐HDAC1 (clone 2E10; Sigma‐Aldrich, Merck Millipore), and anti‐α‐tubulin (T9026; Sigma‐Aldrich, Merck Millipore) antibodies were used. The bands were quantified with Multi Gauge software (FUJIFILM) and analyzed three times with subtraction of the background signal intensity.

### 2.8 RNA isolation and quantitative reverse transcription‐PCR (qRT‐PCR) analysis

Total RNA was extracted with the ISOGEN reagent (Nippon Gene, Toyama, Japan), and cDNA was synthesized with the PrimeScript II 1st strand cDNA Synthesis Kit (Takara Bio, Shiga, Japan). Gene expression was analyzed using the Fast SYBR Green Master Mix with ROX (Roche Diagnostics, Basel, Switzerland) and StepOnePlus Real‐time PCR System (Thermo Fisher Scientific). Primer sequences are shown in Table S1.

### 2.9 RNA‐sequence (RNA‐seq) analysis

Biologically duplicate samples were prepared, and total RNA was extracted from the cells with the RNeasy Mini Kit (Qiagen, Hilden, Germany). Genomic DNA was removed with the RNase‐Free DNase Set (Qiagen), and mRNA was isolated from 10 μg of total RNA. Fragmentation of mRNA, adaptor accession, reverse transcription, amplification, and establishment of cDNA libraries were performed by the Ion Total RNA‐Seq Kit v2 (Thermo Fisher Scientific). Sequencing was performed with the Ion Chef System and Ion Proton, using the Ion PI IC 200 Kit. The sequenced reads were aligned to the human reference sequence (hg19) using Tophat2. Gene expression was calculated with the Cuffdiff function of Cufflinks. Heat map of gene expression was drawn with TM4 MeV. Ontology analysis was performed with the Database for Annotation, Visualization and Integrated Discovery (DAVID) (https://david.ncifcrf.gov). The expression data except for microRNAs and small nucleolar RNAs were also used for gene set enrichment analysis (GSEA).

### 2.10 Statistical analysis

**Fig. 1:**
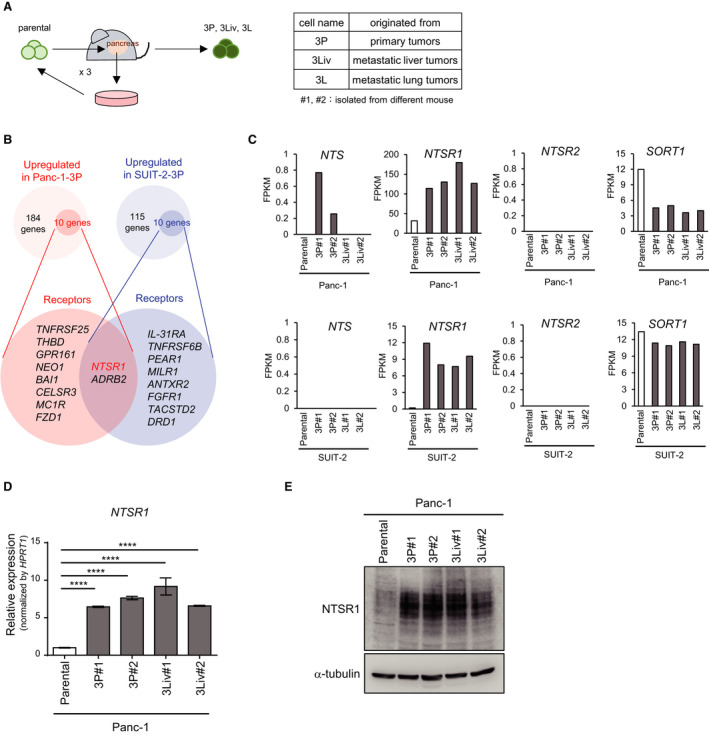
Identification of a key molecule for pancreatic cancer, NTSR1, using orthotopic serial transplantation models. (A) Establishment of pancreatic cancer sublines using an orthotopic tumor model [14]. Parental Panc‐1 cells or parental SUIT‐2 cells were inoculated orthotopically into the pancreas in nude mice. Cells were obtained from the primary tumor and then inoculated into another mouse. This process was repeated three times, and highly malignant cancer sublines were established. More than two sublines were established from each tumor model (#1 and #2) and named 3P cells (from the primary tumor), 3Liv cells (from the metastatic liver tumor), and 3L cells (from the metastatic lung tumor). (B) Screening of candidate genes using RNA‐seq analysis. The genes were purified as follows: (i) the fragments per kilobase of exon per million mapped sequence reads (FPKM) in 3P cells>3 and (ii) increased more than threefold, compared with the FPKM in parental cells. Among them, genes encoding receptors in both SUIT‐2 and Panc‐1 cells were purified. The number of purified genes and their receptor gene symbols are shown in the diagram. (C) Expression of NTS and NTSRs in Panc‐1 cells (top) and SUIT‐2 cells (bottom). Based on RNA‐seq analysis, the expression of NTS and NTSRs in Panc‐1 cells (parental, 3P, and 3Liv) and SUIT‐2 cells (parental, 3P, and 3L) is shown with FPKM. (D) Expression of NTSR1 in Panc‐1 cells. Expression of *NTSR1* mRNA in pancreatic cancer cells was determined by qRT–PCR analysis. Data are presented as mean ± SD. Dunnett’s test is used to compare the multiple samples. ****P < 0.0001. Experiments were repeated more than three times with biologically independent samples. Representative data are shown. (E) The expression of NTSR1 protein in Panc‐1 cells determined by immunoblotting.

**Fig. 2:**
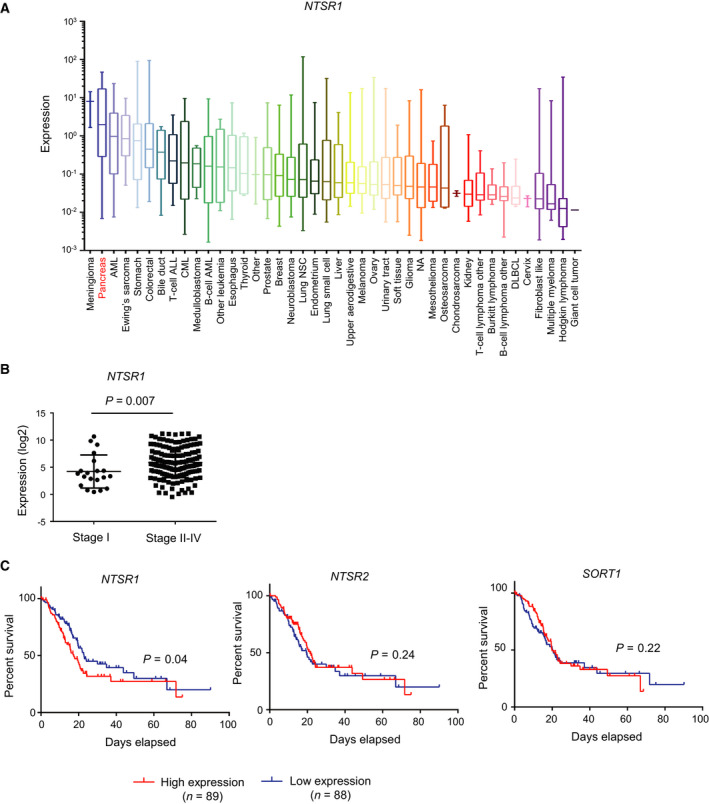
Expression of NTSRs in human pancreatic cancer. (A) The expression of *NTSR1* mRNA in cell lines of various cancers. The data of CCLE were re‐analyzed (RPKM). (B) The expression of *NTSR1* mRNA in primary pancreatic cancer enrolled in the TCGA database. Datasets in the TCGA database (expression median, log2, RSEM) were re‐analyzed. The expression of NTSR1 in stage I and stage II‐IV is shown (stage I; *n* = 21, stage II–IV; *n* = 154). One‐sided Student’s t‐test was used to compare two groups. (C) Kaplan–Meier analysis of overall survival in pancreatic cancer patients enrolled in the TCGA database. Patients were classified into two groups based on the expression of NTSR1, NTSR2, and SORT1 (Z‐scores, RSEM, high expression; *n* = 89, low expression; *n* = 88). Gehan–Breslow–Wilcoxon test was used to evaluate survival curves

**Fig. 3:**
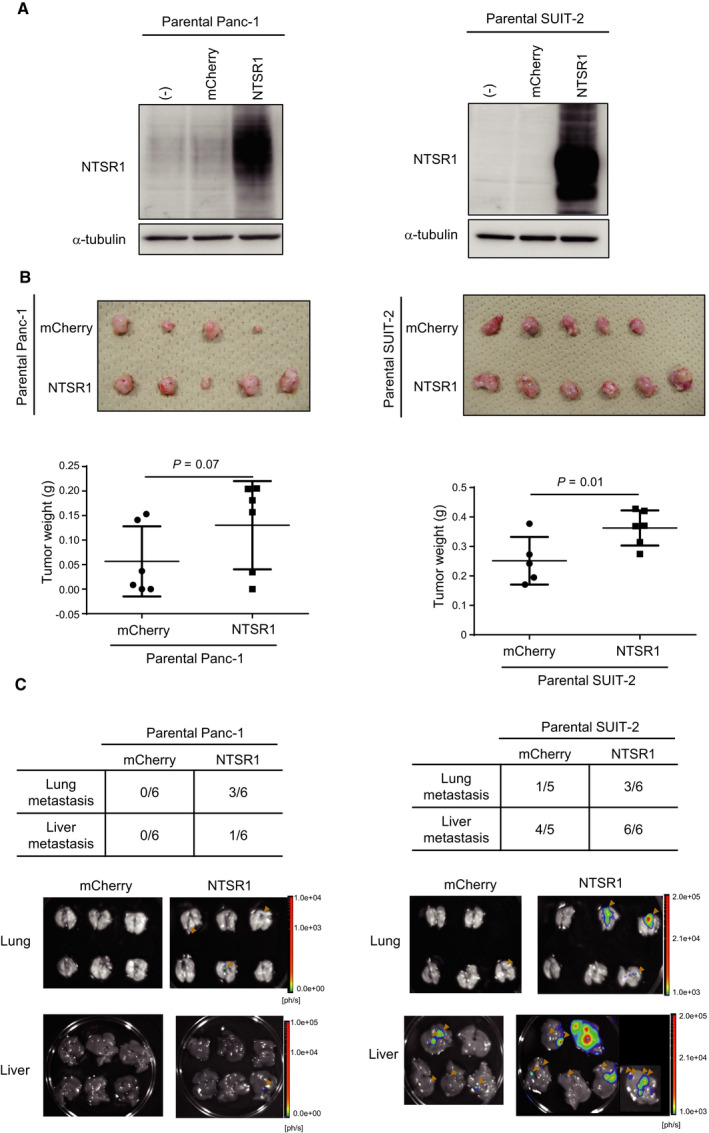
Overexpression of NTSR1 promotes tumorigenicity and metastasis of pancreatic cancer cells. (A) The expression of NTSR1 in NTSR1‐overexpressing cells. NTSR1 or control mCherry was overexpressed in parental Panc‐1 and SUIT‐2 cells using a lentiviral vector system. Increased expression of NTSR1 was confirmed by immunoblotting (left: Panc‐1, right: SUIT‐2). (B) Tumor‐forming ability of NTSR1‐overexpressing parental Panc‐1 or SUIT‐2 cells. mCherry/NTSR1‐overexpressing parental Panc‐1 or SUIT‐2 cells were inoculated into the pancreas orthotopically (*n* = 5–6 per group). Mice bearing Panc‐1‐mCherry or Panc‐1‐NTSR1 cells were euthanized after 2 months. Mice bearing SUIT‐2‐mCherry or SUIT‐2‐NTSR1 cells were euthanized after 1 month. The primary tumors were excised (top; no images are shown for mice without tumor formation). Their weights were measured (bottom). One‐sided Student’s t‐test was used to compare two groups. (C) Metastatic ability of NTSR1‐overexpressing parental Panc‐1 or SUIT‐2 cells. Metastatic tumors in the lungs and livers in mice in (B) were detected by *ex vivo* bioluminescence imaging. The incidence (top) and images (bottom) of lung and liver metastasis are shown. The bioluminescence signals from lung and liver metastatic tumors are indicated with orange arrowheads.

All statistical analyses were performed using GraphPad Prism 6 (GraphPad Software, San Diego, CA, USA). Comparisons between two samples were performed using one‐sided Student’s t‐test, after confirmation of the results of F testing (Fig. 2B, Fig. 3B, Fig. 6D). Gehan–Breslow–Wilcoxon test was used to evaluate survival curves. In qRT–PCR analyses, Dunnett’s test (Fig. 1D, Fig. 5C) or Turkey’s test (Fig. 6C) was used to compare the multiple samples. Significant differences were defined as *P* < 0.05.

## 3 Results

### 3.1 Screening for pancreatic cancer target genes using a mouse orthotopic inoculation model

In our previous study, we established highly malignant pancreatic cancer sublines using serial orthotopic transplantations: 3P cells from primary tumors, 3L cells from metastatic lung tumors, and 3Liv cells from metastatic liver tumors (Fig. 1A). In order to screen for the potential molecular target(s), we extracted the genes expressed in 3P cells. Based on expression data from our previous RNA‐seq analysis (GSE107960), we selected candidate genes that showed high expression levels specifically in 3P cells (Fig. 1B). In this study, we focused upon genes encoding cell surface receptors, which might mediate the interactions between cancer cells and the tumor microenvironment. We identified 10 genes encoding receptors in SUIT‐2‐3P cells and 10 receptor genes in Panc‐1‐3P cells, the expression levels of which were higher than those in the corresponding parental cells. Among them, we found that NTSR1 and adrenergic receptor beta 2 (ADRB2) were commonly upregulated in SUIT‐2‐3P cells and Panc‐1‐3P cells (Fig. 1B). Furthermore, RNA‐seq analysis also showed that the expression of NTSR1 was higher in the sublines, including 3P, 3L, and 3Liv cells, than in the parental cells (Fig. 1C). However, the gene encoding another receptor for NTS, NTSR2, was either not expressed or barely expressed in 3P cells or the other sublines. Although SORT1 (gene for NTSR3) was expressed, its expression level was not higher in 3P, 3L, and 3Liv cells compared with parental cells. Elevation of *NTSR1* mRNA and protein was reproduced in Panc‐1‐3P and Panc‐1‐3Liv cells in an independent qRT–PCR analysis or immunoblotting (Fig. 1D, 1E). Furthermore, the increased expression of *NTSR1* mRNA was observed in SUIT‐2‐3P and SUIT‐2‐3L cells (Fig. S1). These results suggest that the expression of NTSR1 is increased in highly malignant pancreatic cancer sublines and may be important for the acquisition of a malignant phenotype.

### 3.2 Expression of NTSR1 in pancreatic cancer

Next, we examined the clinical significance of NTSR1 expression in pancreatic cancers. According to the Cancer Cell Line Encyclopedia (CCLE), the mRNA expression level of NTSR1 was high in pancreatic cancer cell lines compared with other tumor cell lines (Fig. 2A). In addition, a re‐analysis of the TCGA database on pancreatic cancer showed that the expression of *NTSR1* mRNA was particularly increased in patients with advanced stages of pancreatic cancer (Fig. 2B). Furthermore, patients with high expression of NTSR1 showed a poorer prognosis, compared to patients with low NTSR1 expression (Fig. 2C). In contrast, there was no correlation between the overall survival of pancreatic cancer patients and expression of either NTSR2 or SORT1 (Fig. 2C). These results suggest that NTSR1 may serve as a prognostic marker in patients with pancreatic cancer.

### 3.3 Overexpression of NTSR1 induces highly tumorigenic and metastatic ability in pancreatic cancer cells

The functional role of NTSR1 in pancreatic cancer progression was examined *in vivo* using NTSR1‐overexpressing cells. Since parental Panc‐1 and SUIT‐2 cells showed low expression of NTSR1 (Fig. 1D, E, Fig. 1S), these cells were transfected with NTSR1 or control fluorescent protein (mCherry) using a lentiviral vector system (Fig. 3A). There was no clear difference in the proliferative ability of NTSR1‐overexpressing cells compared with the control mCherry‐expressing cells *in vitro* (Fig. S2). On the contrary, when these cells were inoculated orthotopically into the pancreas of nude mice, we found that NTSR1 promoted primary tumor formation of SUIT‐2 cells significantly and Panc‐1 cells partially *in vivo* (Fig. 3B). When metastatic tumors in the lung and liver were visualized by *ex vivo* bioluminescence imaging, the incidence of lung metastases was increased in mice bearing NTSR1‐overexpressing cells (Fig. 3C). These findings indicate that NTSR1‐mediated signaling plays a tumor‐promoting role in pancreatic cancer.

### 3.4 Various signaling pathways are activated by NTS in pancreatic cancer cells

**Fig. 4:**
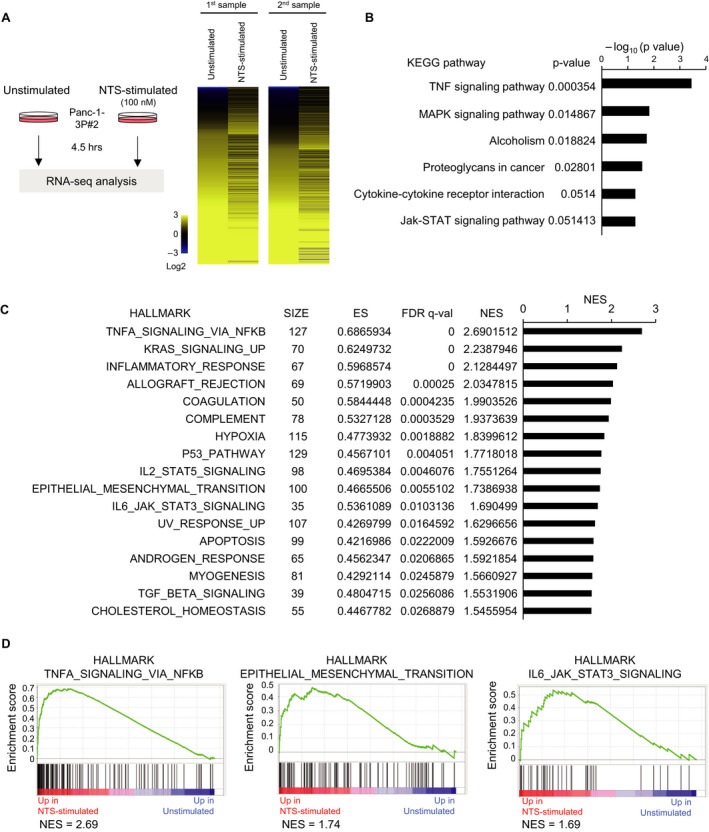
Activation of signaling pathways by NTS in pancreatic cancer cells. (A) A scheme of RNA‐seq analysis of NTS‐stimulated Panc‐1‐3P cells. Panc‐1‐3P#2 cells were stimulated with NTS (100 nm) *in vitro* for 4.5 h and subjected to RNA‐seq analysis using Ion Proton (left). Unstimulated Panc‐1‐3P cells were used as control. Patterns of extracted gene expression are shown in heat map (replicate) (right). (B) Upregulated genes (i) the fragments per kilobase of exon per million mapped sequence reads (FPKM) in stimulated Panc‐1‐3P>2 and (ii) FPKM in NTS‐stimulated Panc‐1‐3P/FPKM in unstimulated Panc‐1‐3P>1.5, 92 genes) were subjected to the Database for Annotation, Visualization and Integrated Discovery (DAVID) analysis. The results from the Kyoto Encyclopedia of Genes and Genome (KEGG) pathway analysis are shown. (C, D) Gene expression data obtained by RNA‐seq analysis were used for gene set enrichment analysis (GSEA). A list of the most enriched hallmark gene sets is presented (C). The number of genes in each set, normalized enrichment score (NES), enrichment score (ES), and false discovery rate (FDR) q‐value is shown (NES>1.5 and FDR *q*‐val < 0.05). Genes of average FPKM (biological duplicate) in either unstimulated or NTS‐stimulated Panc‐1‐3P>5 are used. Representative enrichment plots of the hallmarks in NTS‐stimulated Panc‐1‐3P cells are shown (D).

We investigated the mechanisms underlying NTSR1‐mediated enhancement of pancreatic cell tumorigenicity. Neither the parental cells nor the established pancreatic cancer sublines expressed NTS (Fig. 1C), suggesting that the endogenous NTS/NTSR1 signaling was not activated, at least in an autocrine manner. First, we evaluated the effect of NTS stimulation on the proliferation of pancreatic cancer cells *in vitro*. Exogenous NTS did not promote the proliferation of Panc‐1‐3P cells significantly (Fig. S3). Then, we tried to identify important genes for pancreatic cancer progression among target(s) for NTS/NTSR1 signaling. Panc‐1‐3P cells were stimulated with NTS, the ligand for NTSR1, and then the target genes of NTS/NTSR1 signaling were determined using RNA‐seq analysis, according to the procedure shown in Fig. 4A. The gene expression signatures of NTS‐stimulated Panc‐1‐3P cells were highly different from those in unstimulated Panc‐1‐3P cells (Fig. 4A). In addition, 92 genes were identified as targets of NTS/NTSR1 signaling in Panc‐1‐3P cells, according to the following strategies: (i) the fragments per kilobase of exon per million mapped sequence reads (FPKM) in NTS‐stimulated Panc‐1‐3P>2 and (ii) FPKM in NTS‐stimulated Panc‐1‐3P/ FPKM in unstimulated Panc‐1‐3P>1.5 (Table S2). Then, these target genes were subjected to DAVID analysis. KEGG pathway analysis revealed that NTS activated several intracellular signaling pathways in Panc‐1‐3P cells, such as the mitogen‐activated protein kinase (MAPK), tumor necrosis factor (TNF), and Jak (Janus kinase)‐signal transducer and activator of transcription (STAT) signaling pathways (Fig. 4B). GSEA was also performed with the NTS target genes. The results of enriched hallmark gene sets included the TNF‐nuclear factor (NF)‐κB and interleukin (IL)‐6‐Jak‐STAT3 signaling pathways (Fig. 4C, D). In addition, inflammatory responses and epithelial–mesenchymal transition (EMT) were also induced by NTS stimulation (Fig. 4C, D). These results indicate that NTS activates various signaling pathways that promote the progression of pancreatic cancer.

### 3.5 NTS activates the MAPK and NF‐κB signaling pathways and elevates the expression of target genes

**Fig. 5:**
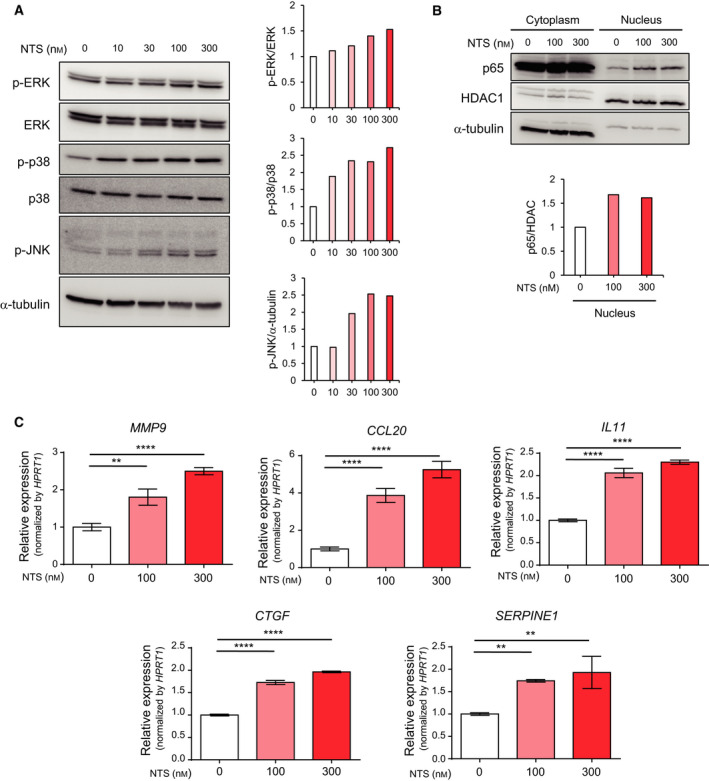
Neurotensin activates the MAPK and NF‐κB signaling pathways and induces the expression of inflammatory genes in pancreatic cancer cells. (A) Activation of the MAPK signaling pathway by NTS. Panc‐1‐3P cells was stimulated with NTS for 5 min. The phosphorylation levels of p44/p42 (ERK), p38, and JNK were determined by immunoblotting (left). Relative expression of phosphorylated proteins was quantified (right). (B) Activation of the NF‐κB signaling pathway by NTS. Panc‐1‐3P cells were stimulated with NTS for 30 min. The amounts of p65 proteins in the cytoplasm and nucleus were determined by immunoblotting (top). Relative expression of p65/HDAC in the nucleus was quantified (bottom). (C) Induction of target genes by NTS. Panc‐1‐3P cells were stimulated with NTS (0, 100, 300 nm) for 4.5 h. Expression levels of *MMP9*, *CCL20*, *IL‐11*, *CTGF,* and *SERPINE1* mRNA in Panc‐1‐3P cells were determined by qRT–PCR analysis. Data are presented as mean ± SD. Dunnett’s test is used to compare the multiple samples. **P < 0.005, ****P < 0.0001. Experiments were repeated more than three times with biologically independent samples. Representative data are shown.

Based on the results of the ontology analysis shown in Fig. 4, we examined the activation of the MAPK and NF‐κB signaling pathways by NTS in Panc‐1‐3P cells by immunoblot analysis. We found that NTS promoted phosphorylation of extracellular signal‐regulated kinase (ERK), p38, and c‐jun N‐terminal kinase (JNK) in a dose‐dependent manner (Fig. 5A, Fig. S4A). In SUIT‐2 cells, phosphorylation of ERK, p38, and JNK was enhanced by NTS stimulation when NTSR1 was introduced (Fig. S4A). Moreover, nuclear localization of p65, an indicator of NF‐κB signaling, was increased by NTS in Panc‐1‐3P cells, as well as SUIT‐2‐NTSR1 cells (Fig. 5B, Fig. S4B).

The induction of NTS/NTSR1 target genes was then validated by qRT–PCR analysis. We found that matrix metalloproteinase (MMP)‐9 was included in the NTS target genes, as previously reported [22]. Furthermore, we detected various other target genes, including connective tissue growth factor (CTGF) and plasminogen activator inhibitor‐1 (PAI‐1/SERPINE1) (Fig. 5C, Table S2), which were reported to be involved in enhanced cellular migration. The pro‐inflammatory cytokines, IL‐11 and C‐C motif chemokine 20 (CCL20), were also highlighted as NTS targets in Panc‐1‐3P cells (Fig. 5C, Table S2). In SUIT‐2‐3P cells, these target genes were highly induced in response to NTS when NTSR1 was introduced (Fig. S5). These results suggest that NTS activates the MAPK and NF‐κB signaling pathways in pancreatic cancer cells, which may be involved in the increased expression of various target genes enhancing cellular migration and inflammation.

### 3.6 Effects of a NTS inhibitor, SR48692, on pancreatic cancer progression

**Fig. 6:**
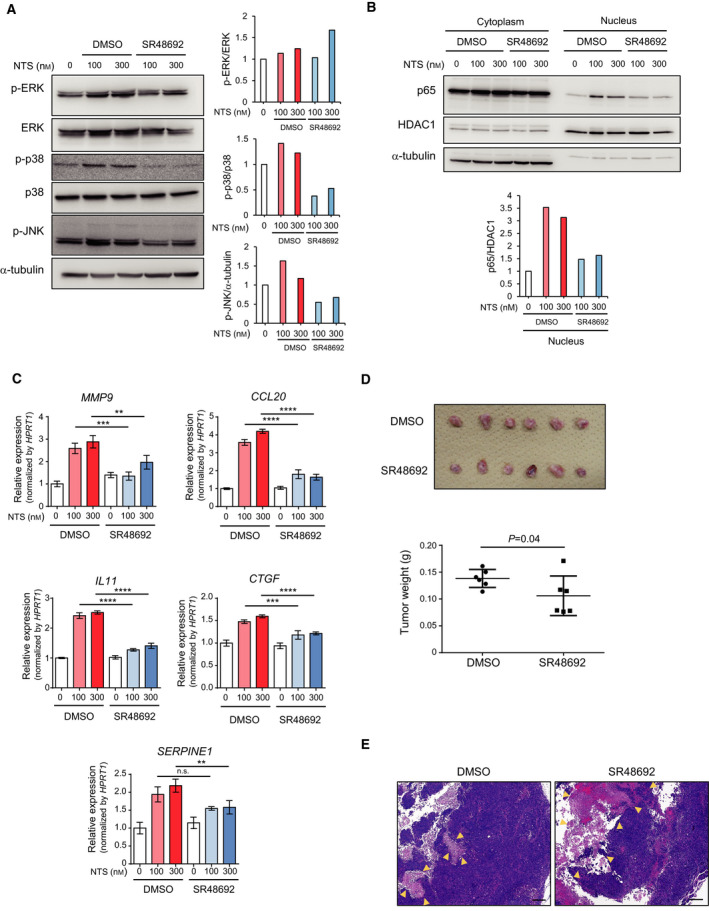
SR48692 suppresses the activation of the MAPK and NF‐κB signaling pathways, induction of target genes, and tumorigenicity of pancreatic cancer cells *in vivo*. (A) The effect of SR48692 on the activation of the MAPK signaling pathways induced by NTS. Panc‐1‐3P cells were pretreated with SR48692 for 10 min and then stimulated with NTS for 5 min. The phosphorylation levels of p44/p42 (ERK), p38, and JNK were determined by immunoblotting (left). Relative expression of phosphorylated proteins was quantified (right). (B) The effect of SR48692 upon activation of the NF‐κB signaling pathway induced by NTS. Panc‐1‐3P cells were pretreated with SR48692 and then stimulated with NTS for 30 min. The amounts of p65 protein in the cytoplasm and nucleus were determined by immunoblotting (top). Relative expression of p65/HDAC in the nucleus was quantified (bottom). (C) The effect of SR48692 on the expression of target genes for NTS. Panc‐1‐3P cells were pretreated with SR48692 for 10 min and then stimulated with NTS for 4.5 h. The expression levels of *MMP9*, *CCL20*, *IL‐11*, *CTGF,* and *SERPINE1* mRNA were determined by qRT–PCR analysis. Data are presented as mean ± SD. Turkey’s test is used to compare the multiple samples. **P < 0.005, ***P < 0.0005, ****P < 0.0001. Experiments were repeated more than three times with biologically independent samples. (D) The effect of SR48692 on the tumorigenicity of the Panc‐1‐3P cells. Panc‐1‐3P cells were inoculated orthotopically. Then, DMSO or SR48692 in PBS was administered intraperitoneally (days −1, 1–7, 10 mg·kg^−1^) (days 8–19, 5 mg·kg^−1^) (n = 6 per group). On day 20, the primary tumors were excised (top) and tumor weights were measured (bottom). One‐sided Student’s *t*‐test was used to compare two groups. (E) Primary tumors in (D) were subjected to hematoxylin and eosin (HE) staining. Representative images are shown. Necrotic areas are indicated by yellow arrowheads. Scale bars = 300 μm.

Next, we analyzed the effects of a small molecular weight NTSR1 antagonist, SR48692 [23], on pancreatic cancer progression. We found that pre‐incubation with SR48692 suppressed the activation of the MAPK signaling pathways by NTS in Panc‐1‐3P cells *in vitro*, although its effect on ERK MAP kinase was not clear (Fig. 6A). Furthermore, SR48692 pretreatment inhibited the nuclear localization of p65 (Fig. 6B). The induction of NTS/NTSR1 target genes, such as MMP‐9, CCL20, IL‐11, CTGF, and PAI‐1/SERPINE1, was also attenuated by the pretreatment with SR48692 (Fig. 6C). Finally, we examined whether SR48692 could inhibit the tumorigenicity of pancreatic cancer cells *in vivo*. The results showed that the administration of SR48692 in mice decreased primary tumor formation in Panc‐1‐3P cell‐bearing mice *in vivo* (Fig. 6D). Moreover, histological examinations demonstrated that primary tumors in SR48692‐treated mice exhibited increased necrotic areas (Fig. 6E). These results suggest that SR48692 potently suppresses pancreatic cancer progression through the inhibition of NTS/NTSR1 signaling.

## 4 Discussion

NTS/NTSR1 signaling has been reported to play multiple roles in the central nervous system and in the digestive tract [24, 25]. NTS binds to two different G‐protein‐coupled receptors (GPCRs) with varying affinities. While it binds to NTSR1 with high affinity, it has a lower affinity for NTSR2 or single transmembranous sorting receptor named NTSR3/Sortilin 1 (SORT1) [26]. NTSR1 is mainly coupled with Gq subunits, which induce phosphatidylinositol (PI) turnover [26]. When the Gq subunit activates phospholipase C (PLC) and activated PLC hydrolyses inositol‐1,4,5‐triphosphate (IP3), intracellular Ca^2+^ is released and protein kinase C (PKC) is activated, resulting in the activation of MAPK signaling pathways [27].

The roles of the NTS/NTSR1 complex have been characterized in cancer cells. Previous studies have shown that NTSR1 is highly expressed in tumor tissues compared with normal tissues and that its expression correlates with a poor prognosis in various kinds of tumors, including glioma, small cell lung carcinoma, endometrial carcinoma, and breast cancer [28, 29, 30, 31, 32]. In the case of pancreatic tumors, increased expression of NTSRs was shown in pancreatic ductal adenocarcinoma and its metastatic liver tumor, as well as in pancreatic intraepithelial neoplasia [15, 16]. However, the receptor(s) functionally involved in this pathway were not fully revealed. We clearly demonstrated that highly malignant pancreatic cancer cells express NTSR1, not NTSR2 and SORT1, as a receptor for NTS. In addition, overexpression of NTSR1 in pancreatic cancer cells resulted in enhanced primary tumor formation and metastasis *in vivo* (Fig. 3). To our knowledge, this is the first report describing a biological function of NTSR1 in pancreatic cancer using mouse tumor models. Increased expression of *NTSR1* mRNA was thought to be a result of a loss of NTSR1 promoter methylation in endometrial adenocarcinoma [28, 33]. However, in the present study, inhibition of DNA methylation with 5‐aza‐2‐deoxycytidine did not increase the expression of NTSR1 in parental Panc‐1 cells as in Panc‐1‐3P cells (data not shown). Analyses using clinical datasets consistently demonstrated that its expression is closely related to the clinical outcome of pancreatic cancer (Fig. 2). Taken together, these findings prompted us to explore the biological function of NTSR1 in pancreatic cancer using mouse models and to identify the target genes of NTS/NTSR1 signaling pathway using genome‐wide gene expression analysis.

Several reports showed that NTS/NTSR1 signaling is involved in the progression of pancreatic cancer. NTS was reported to promote proliferation of pancreatic cancer cells [34, 35, 36, 37, 38, 39]. As a mechanism for it, NTS is thought to activate the ERK and JNK pathways in various pancreatic cancer cells, including Panc‐1 and MiaPACA‐2 [31, 34, 40, 41]. However, downstream signaling pathways for NTS were yet to be fully uncovered in the pancreatic cancer cells. We investigated the activation of the signaling pathways by NTS in the pancreatic cancer sublines by RNA‐seq analysis (Fig. 4). Our data clearly showed that NTS stimulation activated the ERK1/2 and JNK pathways as well as the p38 pathway in Panc‐1‐3P cells. MAPK signaling pathways contribute to cell survival, proliferation, differentiation, and migration of cancer cells [42, 43, 44]. We also demonstrated that NTS stimulation induced TNF superfamily members such as TNF‐α, which in turn activated the NF‐κB signaling pathway in pancreatic cancer cells. It has been widely recognized that NF‐κB is a key transcriptional factor related to pro‐tumorigenic effects, including survival, EMT, metastasis of cancer cells, and inflammation of the tumor microenvironment [45, 46, 47]. Some reports showed that NTS stimulation activated the NF‐κB signaling pathway in normal cells, including preadipocytes and colonic epithelial cells [48, 49, 50]. Although the activation of inflammatory signals by NTS has not been documented in pancreatic cancer, our RNA‐seq analysis showed that NTS activates the NF‐κB and STAT3 inflammatory signaling pathways.

Considering the various biological roles of NTS/NTSR1 signaling, it is important to identify the NTS/NTSR1 target gene(s). In previous studies, NTS stimulation induced the production of pro‐inflammatory cytokines, such as IL‐1, IL‐6, and IL‐8, in macrophages, adipose tissues, and colonic epithelial cells [26, 48, 49, 50]. Similar to normal cells, several reports also showed that NTS stimulation induced IL‐8 production in colon and pancreatic cancer cells [51, 52]. A recent study identified downstream NTS/NTSR1 targets which were important for neuroendocrine differentiation of CK8^+^/CK14^+^ prostate cancer cells [53]. In the present study, we identified the NTS/NTSR1 target genes in pancreatic cancer cells by a genome‐wide RNA‐seq analysis. The expression of MMP‐9 was upregulated in Panc‐1‐3P cells and NTSR1‐overexpressing SUIT‐2 cells, which was in accordance with a previous report in gastric cancer cells [22]. In addition to MMP‐9, various kinds of secreted proteins were identified as targets for NTS/NTSR1 in pancreatic cancers. SERPINE1 and CTGF were produced under NTS stimulation, probably endowing cancer cells with invasive and metastatic abilities. Moreover, pancreatic cancer cells secrete IL‐11 and CCL20 in response to NTS stimulation, which in turn evoke inflammatory responses in the tumor microenvironments. Although further studies are needed, our findings suggest that NTS/NTSR1 signaling contributes to the acquisition of a malignant phenotype by pancreatic cancer cells through the induction of EMT and inflammation.

The pharmacological effect of inhibition of NTSR1 signaling using siRNAs or SR48692 has been already estimated in a wide variety of cancers, such as lung cancer, colon cancer, ovarian cancer, melanoma, and glioma [54, 55, 56, 57, 58, 59]. In pancreatic cancer, previous studies demonstrated that SR48692 attenuated the proliferation and tumorigenicity of pancreatic cancer cells induced by exogenous stimulation with NTS [36, 60]. We showed that SR48692 inhibits tumor formation of pancreatic cancer cells *in vivo* (Fig. 6). Importantly, we demonstrated that the expression of genes related to the invasive abilities of cancer cells was also attenuated by SR48692 (Fig. 6). Our results thus validated proof of concept for NTS/NTSR1‐targeting strategy in pancreatic cancer treatment.

## 5 Conclusions

We showed that NTSR1 is specifically expressed in highly malignant pancreatic cancer sublines, which was also observed in clinical datasets of pancreatic cancers. Overexpression of NTSR1 in pancreatic cancer cells promoted tumorigenicity and metastatic ability *in vivo*, suggesting a pro‐tumorigenic role for NTS/NTSR1 signaling. In addition, RNA‐seq analysis revealed the activation of various signaling pathways and the induction of many new target genes by NTS in pancreatic cancer cells, which were suppressed by SR48692. We also revealed that the administration of SR48692 attenuated the tumorigenicity of pancreatic cancer cells *in vivo*. These findings suggested that NTSR1 may be an ideal target for pancreatic cancer treatment.

## Author contributions

KT, SE, and K. Miyazono designed the study. KT, K. Miyauchi, and YM performed the experiments. KT, SE, K. Miyazawa, and K. Miyazono analyzed the data and wrote the manuscript. All authors discussed the results and commented on the manuscript.

## Conflict of interest

K. Miyazono and S.E. were partly supported by Eisai, Co., Ltd. The remaining authors declare no conflict of interest.

### Peer Review

The peer review history for this article is available at https://publons.com/publon/10.1002/1878‐0261.12815.

## Supporting information


**Fig. S1.** The expression of NTSR1 in SUIT‐2 cells.

**Fig. S2.** Cell proliferation of NTSR1‐overexpressing cells.

**Fig. S3.** The effect of NTS on proliferation of Panc‐1‐3P cells.

**Fig. S4.** NTS activates the MAPK and NF‐κB signaling pathways and induces expression of inflammatory genes in NTSR1‐overexpressing parental SUIT‐2 cells.

**Fig. S5.** Induction of the target genes in NTSR1‐overexpressing parental SUIT‐2 cells.

**Tables S1.** Primer sequences for qRT‐PCR analyses.
**Tables S2.** Genes upregulated by NTS in Panc‐1‐3P cells.

## Data Availability

Raw and processed RNA‐seq data are available at GEO (GSE107960 and GSE147159).
