# Supplementary figures and images for: Neurotensin receptor 1 signaling promotes pancreatic cancer progression
Source: Mol Oncol. 2020 Nov 20;15(1):151–66. doi: 10.1002/1878-0261.12815 (PMC7782081; doi:10.1002/1878-0261.12815)

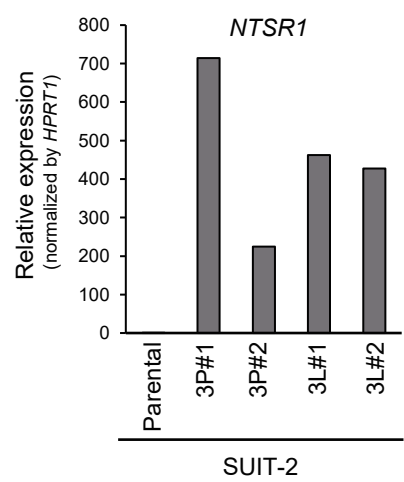

Supplement: Supplementary file 1 — Fig. S1. The expression of NTSR1 in SUIT‐2 cells. [file MOL2-15-151-s001.pdf]

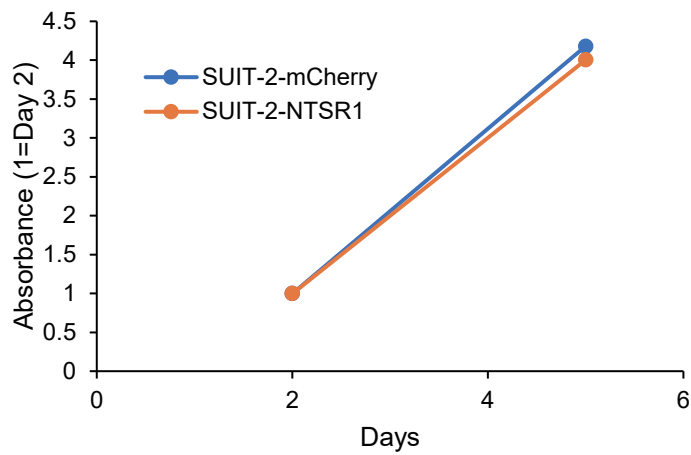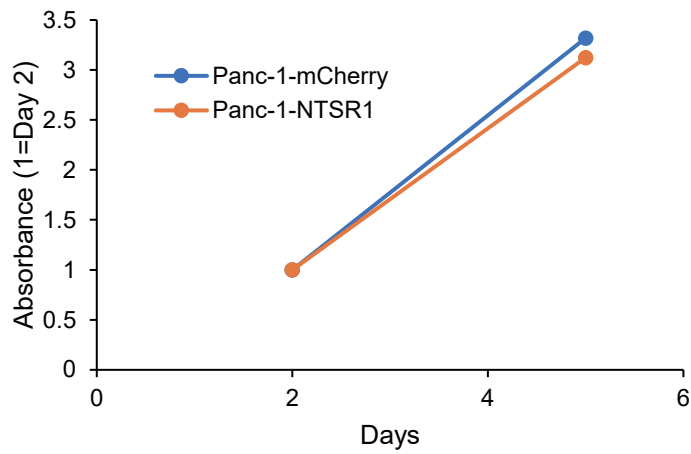

Supplement: Supplementary file 2 — Fig. S2. Cell proliferation of NTSR1‐overexpressing cells. [file MOL2-15-151-s002.pdf]

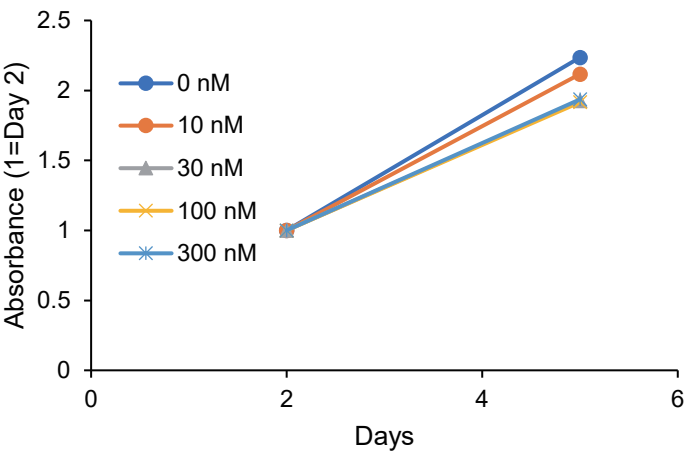

Supplement: Supplementary file 3 — Fig. S3. The effect of NTS on proliferation of Panc‐1‐3P cells. [file MOL2-15-151-s003.pdf]

A

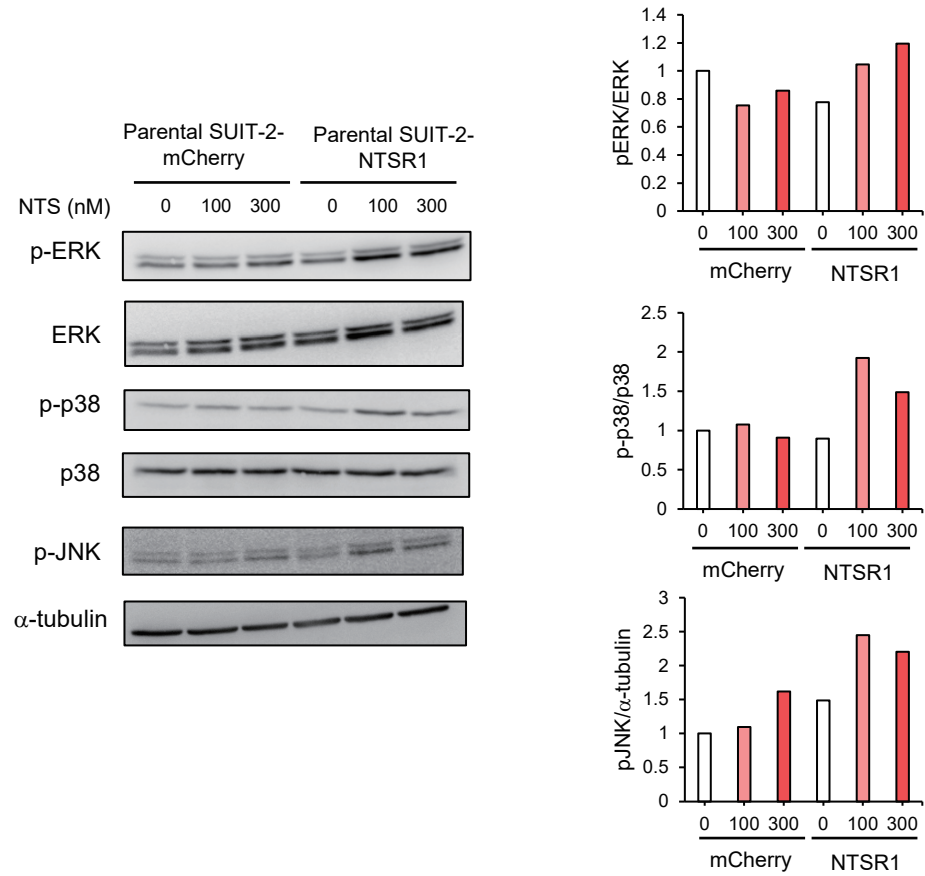

B

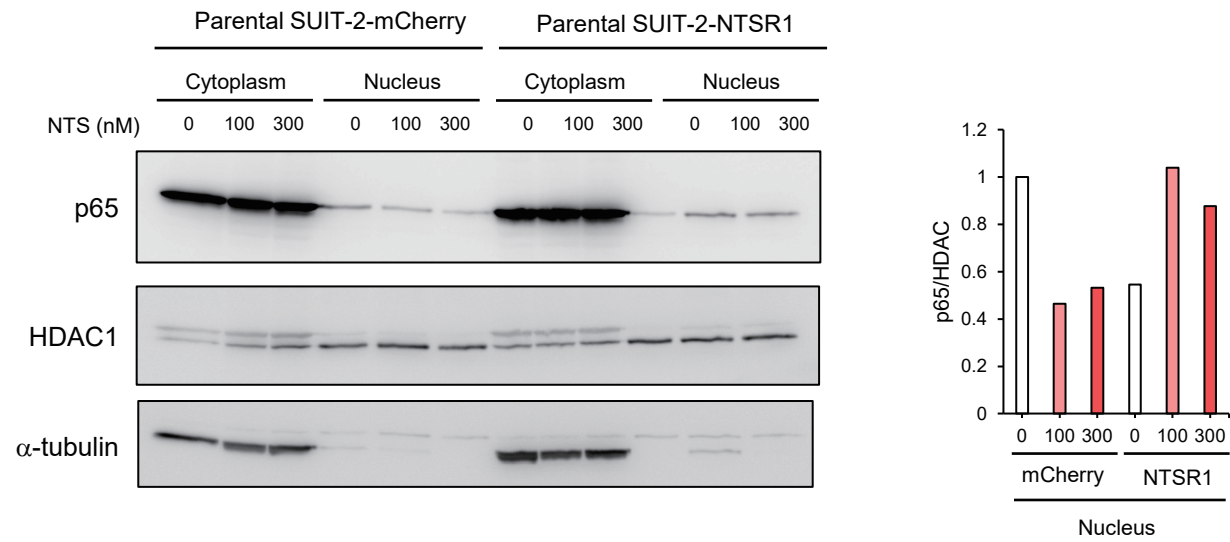

Supplement: Supplementary file 4 — Fig. S4. NTS activates the MAPK and NF‐κB signaling pathways and induces expression of inflammatory genes in NTSR1‐overexpressing parental SUIT‐2 cells. [file MOL2-15-151-s004.pdf]

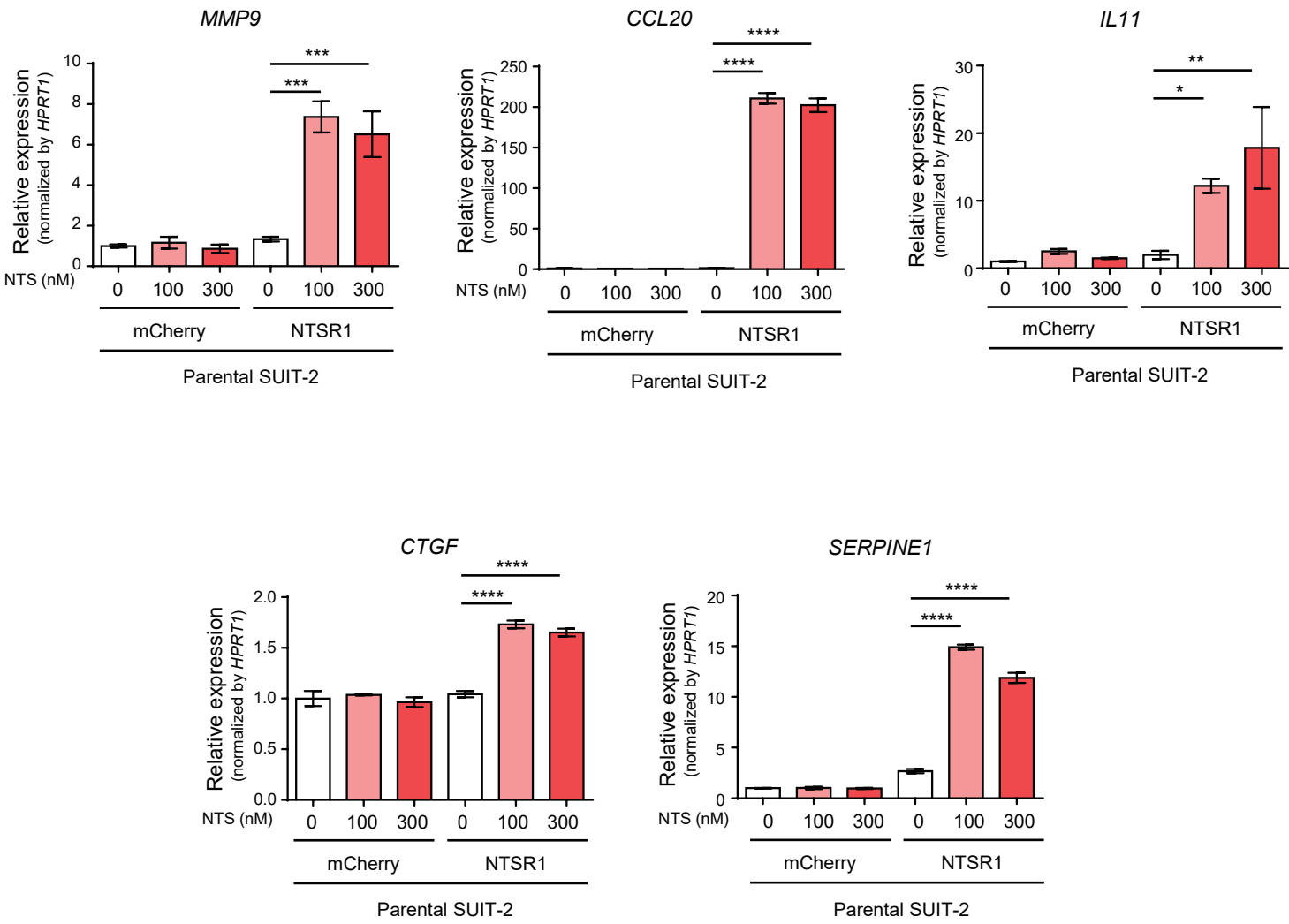

Supplement: Supplementary file 5 — Fig. S5. Induction of the target genes in NTSR1‐overexpressing parental SUIT‐2 cells. [file MOL2-15-151-s005.pdf]
