# Supplementary material for: Neurotensin receptor 1 signaling promotes pancreatic cancer progression
Source: Mol Oncol. 2020 Nov 20;15(1):151–66. doi: 10.1002/1878-0261.12815 (PMC7782081; doi:10.1002/1878-0261.12815)
Supplement: Supplementary file 6 — Tables S1. Primer sequences for qRT‐PCR analyses. Tables S2. Genes upregulated by NTS in Panc‐1‐3P cells. [file MOL2-15-151-s006.docx]

**Supporting Information**

**Supplementary Table S1. Primer sequences for qRT-PCR analyses.**

| Gene | Forward/Reverse | Sequence (5’ to 3’) |
| --- | --- | --- |
| hNTSR1 | Forward | ACCGTCAAGGTCGTCATACAG |
|  | Reverse | TGATGGTGTTCAGGACCGAGA |
| hHPRT1 | Forward | TTTGCTTTCCTTGGTCAGGC |
|  | Reverse | GCTTGCGACCTTGACCATCT |
| hMMP9 | Forward | CCTGGGCAGATTCCAAACCT |
|  | Reverse | GCAAGTCTTCCGAGTAGTTTTG |
| hCTGF | Forward | GGCCTTGCGAAGCTGACC |
|  | Reverse | GGATGCACTTTTTGCCCTTCT |
| hIL11 | Forward | AGCGAGTGGATCACTGAAGTCC |
|  | Reverse | TTGCCATGTCTCGCAGGC |
| hCCL20 | Forward | TGCTGTACCAAGAGTTTGCTC |
|  | Reverse | CGCACACAGACAACTTTTTCTTT |
| hSERPINE1 | Forward | GGCTGACTTCACGAGTCTTTCA |
|  | Reverse | ATGCGGGCTGAGACTATGACA |

**Supplementary Table S2. Genes upregulated by NTS in Panc-1-3P cells.**

Panc-1-3P cells stimulated with NTS for 4.5 h, FPKM > 2, fold induction >1.5, 92 genes

| *HIST1H2AD* | *NAV3* | *MCART1* |
| --- | --- | --- |
| *NR4A2* | *ZNF266* | *ERCC1* |
| *NPPC* | *MMP9* | *GIPR* |
| *CCL20* | *EGR1* | *RPL13AP20* |
| *NR4A1* | *ZSWIM4* | *LIF* |
| *IL11* | *STC1* | *PIK3CD* |
| *CITED1* | *DGKD* | *SPRY4* |
| *DUSP5* | *EIF2C2* | *MAP2K3* |
| *CCDC75* | *S100A3* | *TM4SF1* |
| *KDM6B* | *MAFF* | *SERINC5* |
| *CNN1* | *SERPINE1* | *BCL10* |
| *PLAUR* | *C17orf96* | *CTGF* |
| *FOSB* | *HBEGF* | *ARSI* |
| *SOX17* | *SEMA7A* | *TMEM2* |
| *FGF1* | *RAB3B* | *ABHD11-AS1* |
| *ST3GAL1* | *HIVEP2* | *PDGFB* |
| *IQCJ-SCHIP1* | *PPM1J* | *HIST1H3C* |
| *LOC146880* | *SPHK1* | *GLIPR1* |
| *EMP1* | *MARCH4* | *UHRF1BP1L* |
| *ARC* | *HDAC9* | *PAQR5* |
| *LOC100287559* | *ELL2* | *MT1DP* |
| *THBD* | *LOC400043* | *TNFRSF12A* |
| *SLC35E4* | *DOT1L* | *IER3* |
| *FBXO32* | *PIM3* | *SOCS1* |
| *DLL4* | *TRIB1* | *ADRB2* |
| *TFPI2* | *HIST1H4E* | *LRCH1* |
| *C12orf47* | *TBC1D4* | *SH3D21* |
| *LOC100652730* | *KIAA0040* | *FOXD1* |
| *EGR4* | *CDC37L1* | *FLNC* |
| *CLCF1* | *TTC9B* | *SNCB* |
| *FAM174A* | *BIRC3* |  |

**Supplementary Figure S1. The expression of NTSR1 in SUIT-2 cells.**

Expression of NTSR1 mRNA in SUIT-2 cells was determined by qRT-PCR analysis. Data are presented as mean (duplicate).

**Supplementary Figure S2. Cell proliferation of NTSR1-overexpressing cells.**

Cell proliferation assay of NTSR1 or control mCherry-overexpressing pancreatic cancer cells. Parental SUIT-2 or Panc-1 cells overexpressing NTSR1 or control mCherry were seeded into 96-well plates and cultured for 2–5 d. Relative absorbance (450–595 nm) at the indicated days is shown.

**Supplementary Figure S3. The effect of NTS on proliferation of Panc-1-3P cells.**

Cell proliferation assay of pancreatic cancer cells. Panc-1-3P cells were seeded into 96-well plates and treated with NTS at indicated concentrations. Cells were cultured for 2–5 d. Relative absorbance (450–595 nm) at the indicated days is shown.

**Supplementary Figure S4. NTS activates the MAPK and NF-κB signaling pathways and induces expression of inflammatory genes in NTSR1-overexpressing parental SUIT-2 cells.**

(A) Activation of the MAPK signaling pathway by NTS. Parental SUIT-2 cells overexpressing NTSR1 or control were stimulated with NTS for 5 min. The phosphorylation levels of p44/p42 (ERK), p38 and JNK were determined by immunoblotting (left). Relative expression of phosphorylated proteins was quantified (right). (B) Activation of the NF-κB signaling pathway by NTS. Parental SUIT-2 cells overexpressing NTSR1 or control mCherry were stimulated with NTS for 30 min. The amounts of p65 proteins in the cytoplasm and nucleus were determined by immunoblotting (left). Relative expression of p65/HDAC in the nucleus was quantified (right). **Supplementary Figure S5. Induction of the target genes in NTSR1-overexpressing parental SUIT-2 cells.**

Induction of the target genes by NTS. Parental SUIT-2 cells overexpressing NTSR1 or control mCherry were stimulated with NTS (0, 100, 300 nM) for 4.5 h. Expression levels of MMP9, CCL20, IL11, CTGF and SERPINE1 mRNA were determined by qRT-PCR analysis. Data are presented as mean ± SD. Turkey’s test is used to compare the multiple samples. **P < 0.05,* ***P < 0.005, ***P < 0.0005, ****P < 0.0001.* Experiments were repeated more than three times with biologically-independent samples.
